# Regulatory T Cells as an Escape Mechanism to the Immune Response in *Taenia crassiceps* Infection

**DOI:** 10.3389/fcimb.2021.630583

**Published:** 2021-04-13

**Authors:** Laura Adalid-Peralta, Alexander Lopez-Roblero, Cynthia Camacho-Vázquez, Marisol Nájera-Ocampo, Adrián Guevara-Salinas, Nataly Ruiz-Monroy, Marlene Melo-Salas, Valeria Morales-Ruiz, Dina López-Recinos, Edgar Ortiz-Hernández, Jocelyne Demengeot, Joel A. Vazquez-Perez, Asiel Arce-Sillas, Sandra Gomez-Fuentes, Robert Michael Evans Parkhouse, Gladis Fragoso, Edda Sciutto, Edgar E. Sevilla-Reyes

**Affiliations:** ^1^ Unidad Periférica para el Estudio de la Neuroinflamación en Patologías Neurológicas del Instituto de Investigaciones Biomédicas de la UNAM en el Instituto Nacional de Neurología y Neurocirugía México, Ciudad de México, Mexico; ^2^ Instituto Nacional de Neurología y Neurocirugía, Ciudad de México, Mexico; ^3^ Facultad de Ciencias Químicas, Universidad Autónoma de Chiapas, Tapachula, Mexico; ^4^ Instituto Gulbenkian de Ciência, Oeiras, Portugal; ^5^ Departamento de Investigación en Enfermedades Infecciosas, Instituto Nacional de Enfermedades Respiratorias Ismael Cosío Villegas, Tlalpan, Mexico; ^6^ Departamento de Inmunología, Instituto de Investigaciones Biomédicas, Universidad Nacional Autónoma de México, Ciudad de México, Mexico

**Keywords:** Tregs, parasites, *Taenia crassiceps*, cysticercosis, susceptibility

## Abstract

Murine cysticercosis by *Taenia crassiceps* is a model for human neurocysticercosis. Genetic and/or immune differences may underlie the higher susceptibility to infection in BALB/cAnN with respect to C57BL/6 mice. T regulatory cells (Tregs) could mediate the escape of *T. crassiceps* from the host immunity. This study is aimed to investigate the role of Tregs in *T. crassiceps* establishment in susceptible and non-susceptible mouse strains. Treg and effector cells were quantified in lymphoid organs before infection and 5, 30, 90, and 130 days post-infection. The proliferative response post-infection was characterized *in vitro*. The expression of regulatory and inflammatory molecules was assessed on days 5 and 30 post-infection. Depletion assays were performed to assess Treg functionality. Significantly higher Treg percentages were observed in BALB/cAnN mice, while increased percentages of activated CD127+ cells were found in C57BL/6 mice. The proliferative response was suppressed in susceptible mice, and Treg proliferation occurred only in susceptible mice. Treg-mediated suppression mechanisms may include IL-10 and TGFβ secretion, granzyme- and perforin-mediated cytolysis, metabolic disruption, and cell-to-cell contact. Tregs are functional in BALB/cAnN mice. Therefore Tregs could be allowing parasite establishment and survival in susceptible mice but could play a homeostatic role in non-susceptible strains.

## Introduction

Murine intraperitoneal cysticercosis by *Taenia crassiceps* has been extensively used to study the influence of the host’s genetic background, sex factors, and immunity on the reproduction of infecting cysticerci. Previous studies demonstrated that BALB/cAnN mice are more susceptible to parasite infection than C57BL/6 mice (Fragoso et al., 1996; Fragoso et al., 2008). Genetic differences between both mouse strains are widely documented, such as the background H-2 gene complex (Sciutto et al., 1991), the gene coding for the Qa-2 protein (Fragoso et al., 1996; Fragoso et al., 1998) and the locus called Tccr1 (*T. crassiceps* cysticercosis restrictive locus 1), associated to their distinctive phenotype (Ramirez-Aquino et al., 2011).

Despite host infection restrictions, several strategies are used by cysticerci to evade the host immune response. During their establishment, *T. crassiceps* cysticerci induce a Th1-type immune response, whilst late-stage cysticerci promote a Th2-type response that favors parasite persistence in the host (Terrazas et al., 1998; Peón et al., 2013). The parasite secretes molecules to evade certain components of the complement system, like paramyosin, which inhibits C1q (Laclette et al., 1992), and taeniastatin, which inhibits the factor D and the C3 esterase (White et al., 1992; White et al., 1997). Cysticerci also secrete a metallo-aminopeptidase that degrades cytokines and cysteine-proteases to degrade antibodies (White et al., 1992; White et al., 1997; Baig et al., 2005). Other strategies include antibody-coating (McManus and Lamsam, 1990; Flores-Bautista et al., 2018) and a suppression of the specific proliferative immune response by limiting IL-2, a growth factor of proliferating effector cells (Sciutto et al., 1995; Tato et al., 1995; Hernández-Mendoza et al., 2005; Peón et al., 2013). T regulatory cells (Tregs) may play a critical role in this specific suppression of immunity. Evidences of increased Tregs levels in peripheral blood and the cerebrospinal fluid from human neurocysticercosis patients, which negatively correlate with lymphocyte activation, support this possibility (Adalid-Peralta et al., 2012). In fact, Treg induction favors the establishment and survival of parasites like *Heligmosomoides polygyrus* (Grainger et al., 2010), *Echinococcus multilocularis* (Nono et al., 2020) and *Plasmodium* sp. (Hisaeda et al., 2004).

Tregs are known to exert their immunomodulatory effects by several mechanisms. Tregs produce suppressor cytokines like IL-10, TGFβ, and IL-35; they are also are able to secrete granzyme and perforin, inducing cytolysis in effector cells (Vignali et al., 2008). Tregs inhibit the proliferation of effector T cells due to their high constitutive expression of CD25 (the IL-2 receptor α-chain), which grants them with a higher affinity to IL-2 than that exhibited by effector cells; thus, Tregs capture this growth factor, inducing death in T effector cells by cytokine deprivation (Vignali et al., 2008). Tregs may cause metabolic disruption by cyclic AMP (cAMP)-mediated inhibition, and by CD39- and/or CD73- generated adenosine receptor 2A (A2AR)-mediated immunosuppression (Vignali et al., 2008). Tregs may engage in a cell-to-cell interaction with dendritic cells through CTLA-4 and LAG-3; this interaction promotes a tolerogenic phenotype in dendritic cells (DCs) (Vignali et al., 2008). The effects of these suppressive mechanisms on the response of effector cells during parasite infection could be favoring the establishment and persistence of the parasite in the host.

Thus, a successful infection by *T. crassiceps* in an immunocompetent host depends on several parasite-host molecular interactions. In this work, we studied the role of Tregs in *T. crassiceps* infection to BALB/cAnN (susceptible) and C57BL/6 (resistant) mice, and the contribution of this cell population to parasitism.

## Materials and Methods

### Ethics Approval and Accordance

All experimental protocols were approved by the Scientific Committee and the Internal Committee for the Use and Care of Laboratory Animals (Permit No. 14/16) of the Instituto Nacional de Neurología y Neurocirugía. All methods were carried out in accordance with the guidelines and regulations in the Mexican Official Standard NOM 062 ZOO 1999.

### Mice

Female BALB/cAnN and C57BL/6 mice, 4-5 weeks-old, were kept in the vivarium at the Instituto Nacional de Neurología y Neurocirugía. All animals were kept and used in accordance to the Mexican official standard NOM-062-ZOO-1999.

In total, 48 mice of each strain were used to determine the percentage and proliferative response of classical Treg (CD4+CD25+FOXP3+), naïve-like (CD4+CD25−Foxp3−CD127+), and activated CD127+ T cell (CD4+CD25+FOXP3−CD127+) populations by flow cytometry (FACs). Uninfected mice were used as controls. Groups of six mice were infected by intraperitoneally inoculating 20, 2-3-mm, non-budding cysticerci per mouse, or mock-inoculated isotonic commercial saline solution (Pisa Farmacéutica, México). Spleen, mesenteric lymph nodes, and peritoneal cells were recovered from six animals per group on days 5, 30, 90, and 130 post-infection. Then, the animals were sacrificed in a CO_2_ chamber. This experiment was performed in duplicate.

### Cell Phenotype

#### T Regulatory Cells

The phenotype of Treg cells was determined using 10^6^ spleen, mesenteric lymph nodes, or peritoneum cells. The following monoclonal antibodies were used for extracellular staining (all purchased from eBioscience, Affymetrix, Santa Clara, CA, unless stated otherwise): rat anti-mouse CD127 FITC (isotype rat IgG2a, κ) or hamster anti-mouse CD3ε FITC (isotype hamster IgG1, κ), rat anti-mouse CD4 Percp-Cy5.5 (isotype rat IgG2a, κ, BD Biosciences), and rat anti-mouse CD25 APC (isotype rat IgG1, κ, BD Biosciences). The cells were incubated with the antibodies for 30 min at 4°C. Then, the cells were left to permeabilize overnight with the Fixation/Permeabilization kit (Affymetrix). The cells were then washed, and intracellular staining was performed with rat anti-mouse Foxp3 PE (isotype rat IgG2a, κ), using the Fixation/Permeabilization kit (Affymetrix). The cells were incubated for 30 min at 4°C, washed, and fixed with (200 µL of 2%) paraformaldehyde in PBS.

#### Naïve/Memory Lymphocytes

To characterize the phenotype of memory, effector, and naïve cells, 10^6^ peritoneum cells were stained with the following antibodies: hamster anti-mouse CD3ϵ FITC (isotype hamster IgG1, κ), rat anti-human/mouse CD44 PE (isotype rat IgG2b, κ), rat anti-mouse CD4 Percp-Cy5.5 (isotype rat IgG2a, κ, BD Biosciences), or rat anti-mouse CD62L APC (isotype rat IgG2a, κ).

##### A) Early and Late Activation Lymphocytes

Early and late lymphocyte activation was determined by staining 10^6^ peritoneum cells with the following antibodies: rat anti-mouse CD38 FITC (isotype rat IgG2a, κ), hamster anti-mouse CD3ϵ PE (isotype hamster IgG1, κ, BD Biosciences), rat anti-mouse CD4 Percp-Cy5.5 (isotype rat IgG2a, κ, BD Biosciences), or rat anti-mouse CD8 Percp-Cy5.5 (isotype rat IgG2a, κ, BD Biosciences), hamster anti-mouse CD69 APC (isotype hamster IgG1, λ).

#### Monocytes, NK and B Lymphocytes

For extracellular staining, 10^6^ peritoneum cells were stained with the following antibodies: hamster anti-mouse CD3ϵ FITC (isotype hamster IgG1, κ), rat anti-mouse CD49b PE (isotype rat IgM, κ, BD Biosciences), rat anti-mouse CD19 Percp-Cy5.5 (isotype rat IgG2a, κ, BD Biosciences), and rat anti-mouse CD138 APC (isotype rat IgG2a, κ, BD Biosciences). In parallel, 10^6^ peritoneum cells were used to determine the monocyte phenotype, using rat anti-mouse CD11b PE (isotype rat IgG2b, κ, BD Biosciences) and rat anti-mouse F4/80 APC (isotype rat IgG2a, κ) antibodies.

Data were read in a FACSCalibur cytometer (Becton Dickinson, Franklin Lakes, NJ) and analyzed with the software CellQuest (Becton Dickinson).

### Flow Cytometry Analysis

#### A) Tregs

To analyze the classic Treg phenotype (CD3+CD4+CD25^hi^FOXP3+) (Hori et al., 2017), the cells were gated according to their forward-side scattering properties; CD3+ and CD4+ double positive cells were selected; then, the gated cells were analyzed for double CD25^hi^ and FOXP3+ expression. To analyze the activated CD127+ T cell (CD4+CD25+Foxp3−CD127^hi^) phenotype, total lymphocytes were first gated according to forward-side scattering properties; CD4+ and CD25^hi^ cells were then selected from these gated cells. A third gate was established according to FOXP3 and CD127 expression. FOXP3-CD127^hi^ events were regarded as activated CD127+ T cells (**Supplementary Figure 1**).

#### B) Naïve/Memory Lymphocytes

Three subpopulations of T lymphocytes were defined: naïve (CD3+CD4+ CD62L^hi^CD44^low^), effector (CD3+CD4+CD62L^low^CD44^hi^), and memory (CD3+CD4+CD62L^hi^CD44^hi^) cells. Total lymphocytes were first gated according to forward-side scattering properties; CD3+ and CD4+ cells were selected from these gated cells. The gated cells were then analyzed with respect to their expression level of CD62L and CD44 (**Supplementary Figure 1**).

#### C) Early and Late Activation Lymphocytes

To analyze CD4 or CD8 activated subpopulations, the cells were gated according to their CD3 expression and forward scattering properties. Total CD3+ cells were analyzed either for double expression of CD4 or CD8 and CD69 (early activation), and CD38 (late activation) (**Supplementary Figure 1**).

#### D) Monocytes, NK, and B Lymphocytes

Total lymphocytes were first gated according to their forward-side scattering properties; B lymphocytes were analyzed for CD19+ expression, and plasma blast cells were analyzed for CD19^low^CD138^hi^ expression. Monocytes were analyzed for the double expression of CD11b and F4/80. NK cells and NKT cells were analyzed for the double expression of CD49b (PanNK)+CD3+ (**Supplementary Figure 1**).

### Lymphoproliferation *In Vitro*

The spleen from infected (as described above) and control mice was recovered and macerated. Red blood cells were lysed with 1% ammonium chloride. About 10^7^ spleen cells suspended in 1 mL of PBS supplemented with 10% bovine fetal serum were intracellularly stained with carboxyfluorescein diacetate succinimidyl ester (CFSE) 5 µM. The cells were incubated for 5 min at 37°C and washed three times with 15 mL of RPMI 10% bovine fetal serum. Then, 10^5^ cells were stimulated with either Concanavalin A (ConA) at a concentration of 5 µg/mL (Sigma, St. Louis, MO) or *T. crassiceps* cyst antigen (10 µg/mL), using 200 µL of RPMI 1640 medium (Gibco BRL, Grand Island, NY) supplemented with antibiotic (1%), 2-mercaptoethanol 50 µM (Sigma-Aldrich), and bovine fetal serum (10%). After 4 days of culture, the cells were harvested, and the total proliferative response was analyzed with a CFSE marker.

Proliferative CD4+ T cells, effector (CD4+CD25^+/low^FOXP3−), and Tregs (CD4+CD25^hi^FOXP3+) were analyzed (**Supplementary Figure 1**). The response percent was calculated as the quotient of the proliferation in infected mice cells divided by the proliferation in controls (average proliferation in the control group on days 5, 30, 90, and 130) and multiplied by 100.

### mRNA Expression Profile of the Immune Response in BALB/cAnN and C57BL/6 Mouse Strains

24 mice of each strain (BALB/cAnN and C57BL/6) were used for this analysis. Mice were distributed into two groups, infected and non-infected. Each mouse in the infected group was intraperitoneally inoculated with 20 2-3-mm, non-budding cysticerci. Six mice from each group were sacrificed in a CO_2_ chamber on days 5 or 30 post-infection, and peritoneum cells were recovered by washing with PBS.

#### A) RNA Extraction

Total RNA was extracted from 10^6^ peritoneal cells with the Qiagen RNeasy mini kit (Hilden, Germany) following manufacturer’s protocols. RNA quantity was determined using the Nanodrop kit (Thermo Scientific, Waltham, MA).

#### B) Reverse Transcription

Two hundred nanograms of total RNA were mixed with 2 μL of random hexamer primers (600 μM) in a total volume of 20 μL and incubated for 10 min at 65°C. A reaction mix for the Transcriptor First Strand cDNA Synthesis Kit (Roche, Foster City, CA) was prepared as follows: 2 μL of dNTP mix (10 mM), 0.5 μL of protector RNase inhibitor (40 U/μL), 0.5 μL of Transcriptor reverse transcriptase (20 U/μL), and 4 μL of 5X reaction buffer. RNA was added to the reaction mix and incubated as follows: 25°C for 10 min, 50°C for 60 min, and 85°C for 5 min.

#### C) Pre-PCR

A pre-PCR amplification mix was prepared with 2.5 μL of TaqMan PreAmp Master Mix (2X) and 0.5 μL of a 35-custom-designed DELTAgene assay mix (Fluidigm, South San Francisco, CA) 500 nM, for a final concentration of 50 nM per assay (**Supplementary Table 1**); 1.25 μL of cDNA were added and taken to a final volume of 5 μL with water. The sample was kept at 95°C for 10 min, followed by 14 cycles of 95°C for 15 s and 60°C for 4 min. After pre-amplification, PCR products were treated with Exonuclease I (New England BioLabs, Ipswich, MA) at 37°C for 30 min and inactivated at 80°C for 15 min and kept at 4°C. Then, the products were diluted 1:5 in water.

#### D) RNA Expression Screening

The expression of 34 genes was determined with a 48.48 IFC system (Fluidigm) by mixing 2.25 μL of Exo I-treated products with 2.5 μL of 2x SsoFast EvaGreen Supermix with Low ROX (Bio-Rad, Berkeley, CA) and 20x DNA Binding Dye Sample Loading Buffer (Fluidigm). Assay mixtures were prepared with 2.5 μL of 2x Assay Loading Reagent (Fluidigm), 2.25 μL of DNA suspension buffer (Fluidigm), and pairs of forward and reverse primers (0.25 μL each) in each IFC outlet. Cycling conditions were: 95°C for 1 min, 30 cycles at 96°C for 5 s and 60°C for 20 s, followed by a dissociation curve from 60 to 95°C at a rate of 1°C for 3 s. Threshold and linear baseline correction was automatically calculated for the entire IFC.

#### E) qPCR Efficiency

To determine the efficiency of each DELTAgene expression assay (**Supplementary Table 1**), serial cDNA dilutions (1:5, 1:25, 1:125, 1:625, 1:3125) from the same source were prepared, aliquoted, and stored for single use. A Real-time PCR analysis software (Fluidigm) was used to analyze the data. Reaction efficiency was determined as usual:

$$E_{gen}=10^{-1/slope}$$

#### F) Gene Expression Analysis

To determine the relative expression of pro-inflammatory and anti-inflammatory genes between groups, the ratio of gene expression was calculated using the differences in expression of the target gene and a reference gene, in mock-infected controls versus infected subjects, using the formula below, correcting for differences in assay efficiency (Arce-Sillas et al., 2019). *E* stands for assay efficiency, *Ref* is reference gene, and *target* is the gene of interest.

$$Ratio=\frac{{\left(E_{t\arg et}\right)}^{\Delta Cq\text{ target(MEAN control-MEAN sample)}}}{{\left(E_{ref}\right)}^{\Delta Cq\text{ target(MEAN control-MEAN sample)}}}$$

Gene expression was measured for *GAPDH, PPIA, ACTB, RPL13A*, and *GUSB* as housekeeping genes candidates. qPCR efficiency was calculated for each gene, and *GAPDH* was chosen as our housekeeping gene standard since it showed the least variation coefficient in control and infected mice.

### Regulatory T Cell Depletion Assays

Since most CD4+ Tregs express the transcription FOXP3 and high levels of the interleukin-2 (IL-2) receptor α-chain (CD25), either 500 μg/mouse of purified anti-mouse CD25 antibody PC61 or 500 μg/mouse of IgG isotype (YCATE, isotype rat, IgG1) as a control were intraperitonially administered to mice to study the relevance of Tregs in parasite establishment. These antibodies were previously demonstrated to effectively deplete Tregs and to bind a different epitope than the antibody used for phenotype determination by flow cytometry (Zelany and Demengeot, 2006; Liu et al., 2010; Maes et al., 2013). On day 2 post-inoculation, each mouse was intraperitoneally infected with 20 cysticerci as described above. The depletion of CD25+ cells was confirmed in two mice randomly selected from each group. Six mice from each group were sacrificed on day 5, 15, or 30 post-infection, and the parasites recovered from the peritoneal cavity were counted. This experiment was performed in duplicate. Tregs and other immune cell phenotypes were collected from the peritoneal cavity and analyzed by flow cytometry. Among CD4+ T cells, the following phenotypes were analyzed: effector (CD3+CD4+CD44^hi^CD62L^low^), memory (CD3+CD4+CD44^hi^CD62L^hi^), naïve (CD3+CD4+CD44^low^CD62L^hi^), early (CD3+CD4+CD69+) and late activation (CD3+CD4+CD38+), and NKT cells (CD3+CD49b+); among T CD8+ cell phenotypes, early (CD3+CD8+CD69+) and late activation (CD3+CD8+CD38+) cells were analyzed; B (CD19+) and plasma cells (CD19−CD138+), as well as monocytes (CD11b+F4/80+), were also analyzed. A group with 6 non-infected mice was included as a control.

### Parasite Counting

The parasites were recovered from the peritoneal cavity of each mouse and placed in a Petri dish. Only non-budding, non-calcified parasites with a size of at least 1-2 mm were counted.

### Treg Depletion in a Murine Melanoma Model (B16)

Female, 4-5 weeks old C57BL/6 mice were randomly distributed into three groups of 6 mice each. One group was intraperitoneally inoculated with 500 μg/mouse of PC61 (anti-CD25) antibody. Another group was inoculated with 500 μg/mouse of IgG isotype (YCATE, isotype rat, IgG1). A control group was inoculated with isotonic saline solution. Two days after inoculation, each mouse was subcutaneously injected with 2 × 10^5^ melanoma B16 cells. Ten days after tumor cells inoculation, tumor size was determined as the product of the longest diameter × perpendicular diameter. Mice were followed up until day 28.

All experiments were performed at least by duplicate, and the plots and figures show the combined results as the mean and standard deviation.

### Statistical Analysis

To analyze differences in the percentage of regulatory T cells and in the immune response after Tregs depletion, either Mann-Whitney U-test or unpaired t-test were used, according to the results of a Shapiro-Wilk normality test.

To analyze the proliferative immune response and regulatory T cell depletion assays, Mann−Whitney U-test was used.

All statistical analyses were performed with the software GraphPad Prism 6 (GraphPad Software Co., California, USA).

## Results

### Differential Percentages of Regulatory T Cells and Activated Cells in *Taenia crassiceps* Infection

**Figure 1:**
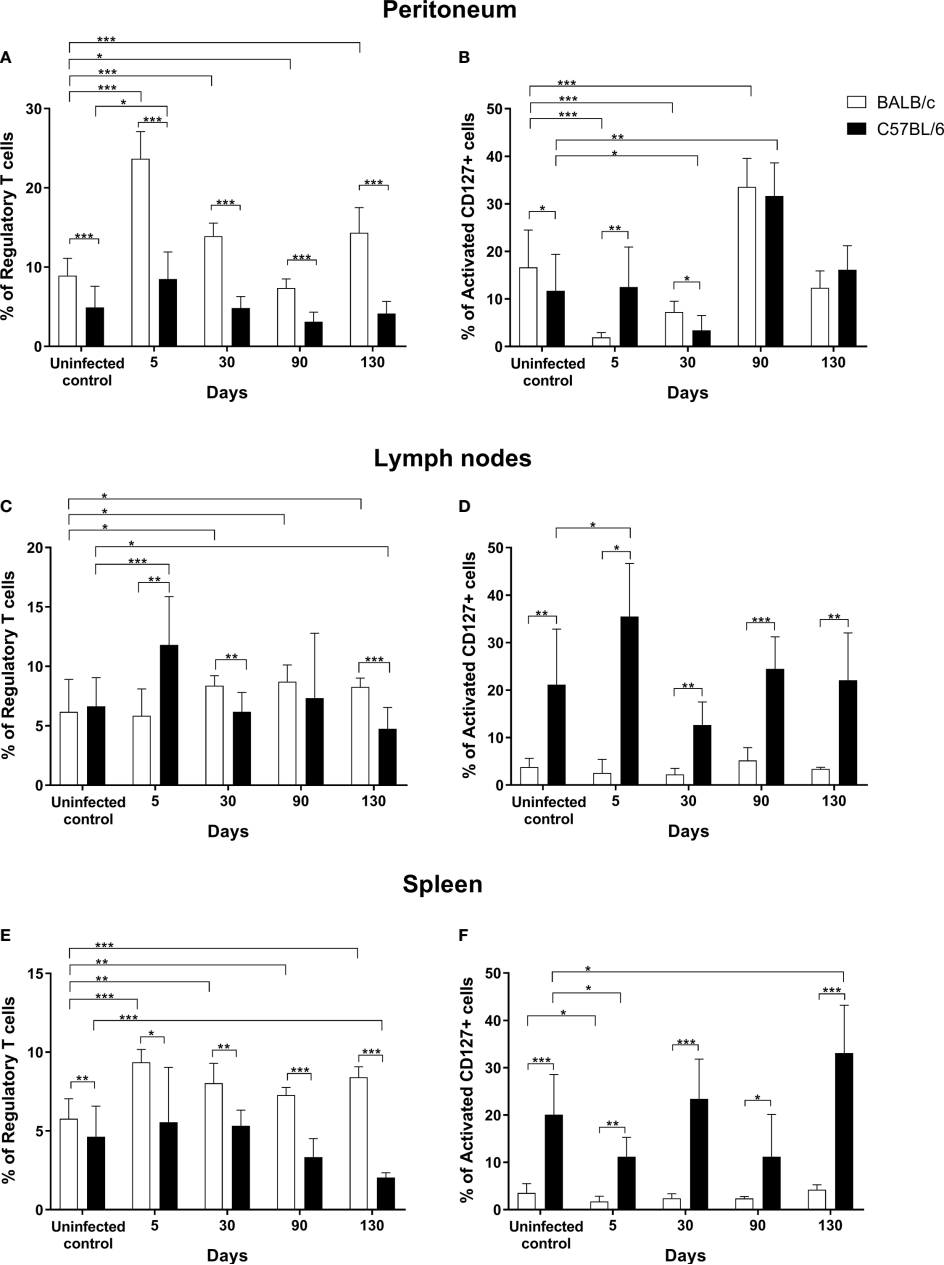
Infection by *Taenia crassiceps* led to increased Treg percentages in the susceptible strain BALB/cAnN (white bars) but not in the resistant strain C57BL/6 (black bars). The percentage of regulatory T cells and activated CD127+ cells is shown in peritoneum **(A, B)**, lymph nodes **(C, D)** and spleen **(E, F)** from BALB/cAnN and C57BL/6 mice. Six infected mice and 6 controls were included for each time point analyzed. Flow cytometry results are reported as a mean and standard deviation (bar graph). Significant differences in cell percentages as determined by the U Mann- Whitney test are shown as *P < 0.05, **P < 0.01, and ***P < 0.005.

The frequency of Tregs (CD4+CD25+FOXP3+) and activated T CD127+ cells (CD4+CD25+FOXP3−CD127^hi^) in infected mice was determined at different times post-infection in the peritoneum and in important secondary lymphoid organs like lymph nodes and spleen. Non-infected mice were included as controls. As shown in **Figure 1A**, Treg percentage in the peritoneum of susceptible, BALB/cAnN mice was significantly increased at early (days 5 and 30) and very late (day 130) infection stages, while on day 90 it was lower than in controls. On the other hand, Treg percentage in the peritoneum of resistant, C57BL/6 mice was significantly higher than in controls only on day 5 post-infection. In a time-course comparison between strains, the BALB/cAnN strain showed significantly higher Treg percentages at all analyzed times.

The percentage of activated CD127+ T cells in the peritoneum of BALB/cAnN mice decreased at early infection stages (days 5 and 30) and increased at late infection stages (day 90), as shown in **Figure 1B**. In C57BL/6 mice, the percentages of activated CD127+ cells were significantly lower on day 30 post-infection but significantly higher on day 90 with respect to controls. In addition, a positive association was found between parasite load and the percentage of activated CD127+ cells (*r* = 0.638, *P* = 0.002) in C57BL/6 mice (plot not shown). In a time-course comparison between strains, significantly higher percentage of activated CD127+ cells were found in C57BL/6 mice on day 5 and lower percentages before infection and on day 30 post-infection. On the other hand, a negative correlation was found between the levels of Tregs and those of activated CD127+ peritoneal cells in susceptible mice (*r* = -0.898, *P* < 0.0001) (**Supplementary Figure 2**). No correlation was found between both populations in C57BL/6, non-susceptible animals.

It should be noted that the peritoneum is the infection site. As shown in the **Supplementary Figure 3**, a significant increase in parasite count was observed in susceptible, BALB/cAnN mice on days 30, 90, and 130 with respect to day 5. Meanwhile, a lower increase in parasite load was observed over time in non-susceptible, C57BL/6 mice, begin significant only between days 5 and 30. Additionally, it is noteworthy that 12-50% of mice in each group of the non-susceptible strain allowed very few parasites to develop or remained uninfected. Furthermore, parasite load was significantly different between both strains at 30, 90 and 130 days (**Supplementary Figure 3**).

In lymph nodes from BALB/cAnN mice, significantly higher Treg percentages with respect to controls were observed on 30, 90, and 130 days post-infection (**Figure 1C**). In contrast, the percentage of Treg and activated CD127+ cells in C57BL/6 mice lymph nodes was increased with respect to controls at early infection stages (day 5) only (**Figures 1C, D**). In a time-course comparison between strains, BALB/cAnN mice showed significantly lower Treg percentages on day 5, but higher Treg percentages on days 30 and 130. Interestingly, C57BL/6 mice showed significantly higher percentage values of activated CD127+ cells at all times than BALB/cAnN.

In spleen cell samples from BALB/cAnN mice, Treg percentage increased significantly with respect to controls at all infection times (**Figure 1E**), while Treg cell percentages in C57BL/6 mice remained constant on days 5, 30, and 90 post-infection, and only on day 130 they were significantly decreased with respect to controls. The percentage of activated CD127+ cells in BALB/cAnN was significantly decreased on day 5 post-infection with respect to controls (**Figure 1F**). In C57BL/6 mice, the percentage of activated CD127+ cells were significantly decreased on day 5 and increased on day 130 post-infection with respect to controls. In a time-course comparison between strains, BALB/cAnN mice showed significantly increased Treg percentages at all times. In contrast, C57BL/6 resistant mice showed significantly increased percentages of activated CD127+ cells at all times.

Additionally, the percentage of total CD4+ T cells and naïve-like cells (CD4+CD25−Foxp3−CD127+) was determined in spleen, lymph nodes, and peritoneum cell samples from both strains (**Supplementary Table 2**). A significant positive correlation was observed between parasite load and CD4+ T cell percentages in spleen samples from C57BL/6 mice (*r* = 0.431, *P* = 0.017) plot not shown.

### Proliferative Immune Response

Cysticerci have been reported to induce an impaired proliferative immune response (Sciutto et al., 1995; Peón et al., 2013). Therefore, the total proliferative response and the percentage of CD4 T proliferative cells (including effector and regulatory T cells) were herein determined in spleen samples from infected and control mice. The results of *T. crassiceps* antigen stimulation and nonspecific stimulation (ConA) in BALB/cAnN and C57BL/6 mice are shown in **Table 1**. Proliferation ratio was calculated by dividing proliferation values in infected mice by proliferation values in controls (the average value in the control group on days 5, 30, 90, and 130).

**Table 1:** Proliferative immune response of spleen cells against non-specific and specific antigens in susceptible (BALB/cAnN) and non-susceptible (C57BL/6) mice.

Day post-infection	Concanavalin A				*Taenia crassiceps* antigen			
	% Total proliferation	% CD4 T cell proliferation	% Effector T cell proliferation	% Treg cells proliferation	% Total proliferation	% CD4 T cell proliferation	% Effector T cell proliferation	% Treg cell proliferation
				**BALB/cAnN**				
**Day 5**	101.89 ± 11.90	72.17* ± 20.37	106.78 ± 4.14	36.05 * ± 41.17	59.48 * ± 11.90	90.98 ± 118.68	103.98 ± 6.37	56.19 ± 137.63
**Day 30**	99.03 ± 4.90	107.05 ± 21.40	100.10 ± 7.00	94.65 ± 68.21	63.10 ± 27.84	176.58 ± 105.40	99.35** ± 3.87	45.64 ± 64.43
**Day 90**	96.67 ± 2.17	105.16 ± 20.11	110.21 ± 6.36	87.38 ± 25.56	88.67 ± 5.30	97.71 ± 38.87	101.09 ± 0.84	111.58** ± 26.4208
**Day 130**	107.79 ± 25.50	133.31 ± 99.20	87.22* ± 6.23	208.49* ± 43.12	91.99 ± 10.43	104.38 ± 28.13	100.57 ± 1.08	64.05 ± 121.31
				**C57BL/6**				
**Day 5**	88.06 ± 35.06	118.27* ± 43.24	100.79 ± 13.95	111.43 * ± 49.00	101.29* ± 32.45	187.88 ± 203.33	84.98 ± 17.38	0.00 ± 0.00
**Day 30**	90.64 ± 24.43	181.62 ± 100.95	92.62 ± 7.36	151.12 ± 69.14	88.04 ± 22.06	288.00 ± 176.98	121.36 ** ± 9.53	0.00 ± 0.00
**Day 90**	84.01 ± 18.28	105.91 ± 25.02	105.17 ± 21.01	112.53 ± 36.73	119.41 ± 114.14	77.78 ± 62.19	99.64 ± 16.59	0.00** ± 0.00
**Day 130**	115.18 ± 34.06	139.68 ± 56.80	98.7469 * ± 9.51	121.85 * ± 73.83	161.77 ± 75.25	182.00 ± 213.91	78.01 ± 44.27	0.00 ± 0.00

$\%\text{ of response}=\frac{Infected\;prolif.}{Mean\;Controls\;prolif.}\times 100$. A 100% response means that the response of the infected mice is equal to that of control mice. Six infected mice and 6 controls were included for each time point analyzed.

Significant differences between BALB/cAnN and C57BL/6 for *P < 0.05 and **P < 0.001.

As expected, the total proliferative immune response to ConA in infected mice was similar to controls (nearly 100%). A comparison between BALB/cAnN and C57BL/6 mouse strains showed that susceptible mice exhibited a significantly lower CD4 and Treg proliferation response on day 5 and a lower effector cell response on day 130, but a significantly higher Treg proliferative response on day 130. With respect to antigen-specific stimulation, susceptible mice exhibited a significantly lower response in total proliferation on day 5 and in effector proliferation on day 30. The CD4 proliferative response was close to controls (nearly 100%). It should be noted that a significant increase in Treg proliferation was observed only in BALB/cAnN mice on day 90.

### Gene Expression Profile of the Immune Response in BALB/cAnN and C57BL/6 Strains During *T. crassiceps* Infection

**Figure 2:**
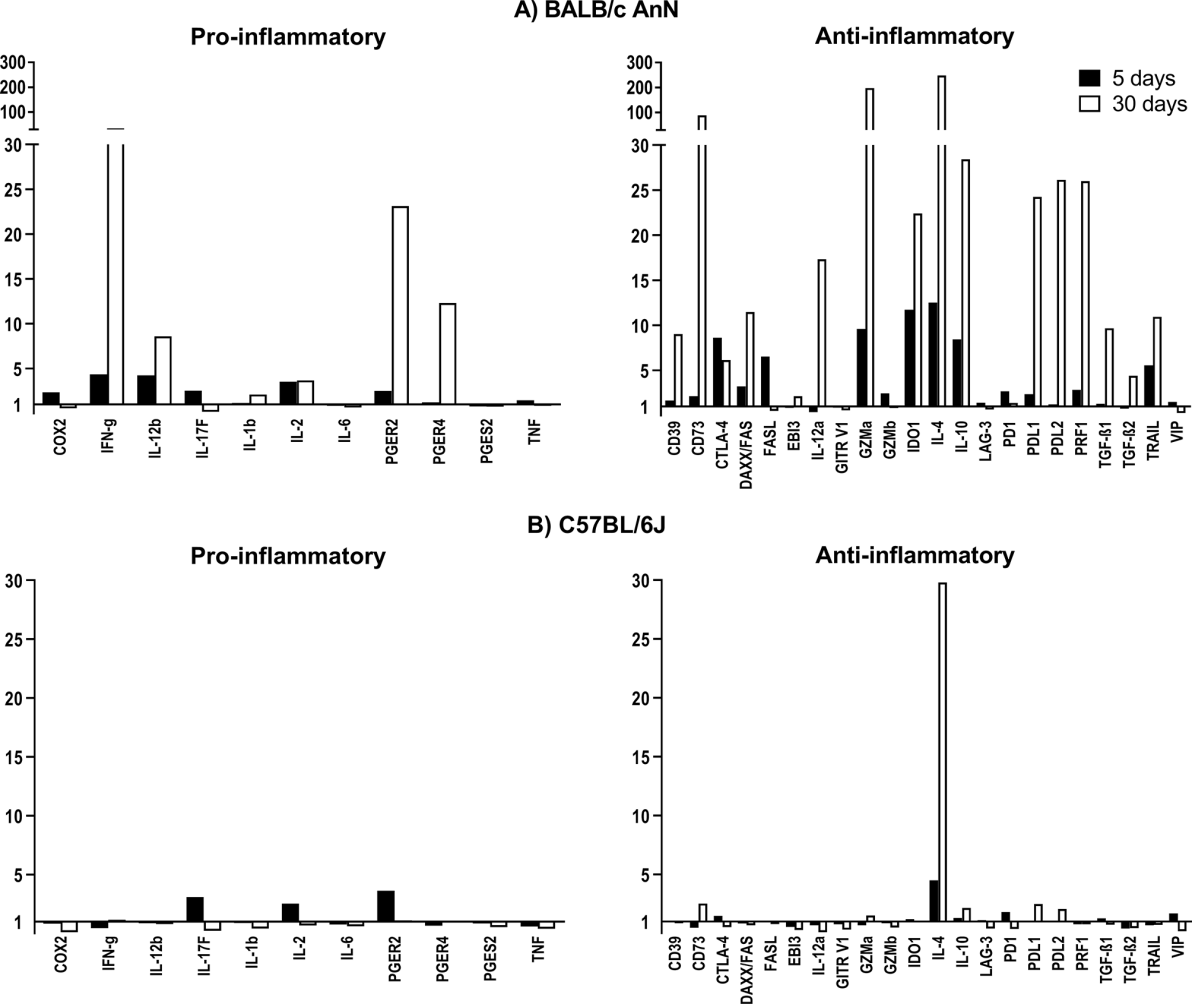
Relative gene expression of molecules involved in the inflammatory and regulatory immune response after infection with *Taenia crassiceps* cysticerci in BALB/cAnN **(A)** and C57BL/6 **(B)** mice on days 5 and 30 post-infection. Six infected mice and 6 controls were included for each time point analyzed.

To characterize the differences between mouse strains in the immune response to *T. crassiceps* infection, the expression of 11 pro-inflammatory and 22 anti-inflammatory genes was assessed in peritoneum cells on days 5 and 30 post-infection. The efficiency of the 34-gene expression assays ranged between 80 and 120% (**Supplementary Table 1**). The relative gene expression normalized to a reference gene (GAPDH) in infected animals with respect to mock-infected controls is shown in **Figure 2**.

In susceptible, BALB/cAnN mice, a 12-fold increase in IL-4 expression was observed on day 5 post-infection, while more modest increases were observed for the expression of IFN-γ and IL-12b. On day 30 post-infection, the expression for these genes was largely increased, along with PGER2 and PGER4. In contrast, the expression of IL-4 and PGER4 in peritoneal cells from C57BL/6 mice was moderately increased on day 5 post-infection, while IL-4 was the only overexpressed gene on day 30 post-infection.

With respect to the gene expression profile of immune suppression molecules, a strain-specific effect was also observed. In BALB/cAnN mice, the expression of CTLA, FASL, GZMa, IDO1, IL-10 and TRAIL was increased on day 5 post-infection, but this profile changed on day 30 post-infection, when FASL expression returned to basal levels, the relative expression of CD39, CD73, FAS, IL-12a, PRF1, PDL1, PDL2, and TGFβ increased, and the expression of IDO1, GZMa, IL-10, and TRAIL remained high. In contrast, no important expression changes of regulatory molecules were found in C57BL/6 mouse cells.

### Regulatory T Cell Depletion Assays

To analyze the role of Treg cells in parasite infection in each mouse strain, Treg cell depletion assays were performed by administering either αCD25 antibody (PC61) or isotype control rat IgG1 (YCATE) to BALB/cAnN and C57BL/6 mice two days before infection. On infection day, two mice from each group were sacrificed, and Treg percentage was determined in peritoneum. As shown in the **Supplementary Figure 4**, a significant decrease in Treg percentage was observed in the peritoneum of all mice treated with αCD25 with respect to controls (isotype-treated).

Treg percentages were determined in the peritoneal cavity of both strains on days 5, 15, and 30 post-infection, as shown in the **Supplementary Figure 5**. In Treg-depleted, non-susceptible mice, Treg percentages increased progressively over time with respect to day 5. In control (isotype-treated) mice, Treg percentages were almost constant. In contrast, in depleted susceptible mice, a significant increase in Treg percentages was observed on days 15 with respect to day 5, and remained increased on day 30. A comparison of Treg percentages between depleted and non-depleted susceptible mice showed a significant increase on day 5.

**Figure 3:**
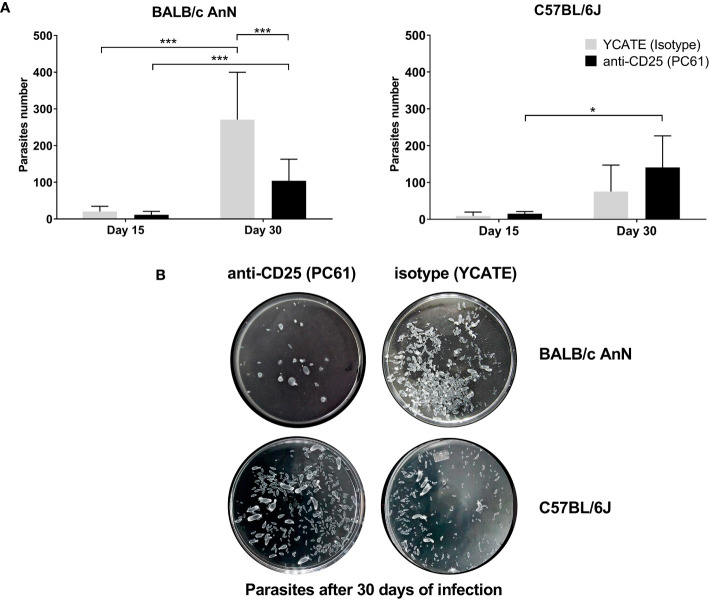
Regulatory T cell depletion decreased parasite load in susceptible mice (BALB/cAnN). Mice treated with either anti-CD25 (PC61) or isotype (YCATE) were infected with *Taenia crassiceps* cysticerci. **(A)** Parasite load was determined in susceptible (BALB/cAnN) and non-susceptible (C57BL/6) mice. Six infected mice and 6 controls were included for each time (15 or 30 days), each condition (isotype or depleted), and each strain. Significant differences were determined by the U Mann-Whitney test. *P < 0.05, **P < 0.01, ***P < 0.005. **(B)** Representative photograph of parasites recovered from one mouse of each strain on day 30 post-infection.

Parasite load was determined on days 15 and 30 post-infection in the peritoneum. As shown in **Figure 3A**, parasite load decreased significantly in depleted susceptible mice (αCD25-treated) with respect to controls (isotype-treated) on day 30. In non-susceptible mice, parasite load was not different between depleted and isotype-treated groups. Representative photographs of parasite load on day 30 post-infection in each mouse strain are shown in **Figure 3B**.

Since Treg depletion did not change with respect to parasite load in C57BL/6 mice, we decided to verify Treg functionality in this strain. Several works have reported that Tregs play a crucial role in suppressing the immune response, favoring tumor cell growth, while Treg depletion is associated to tumor size reduction and even tumor elimination (Onizuka et al., 1999; Nagai et al., 2004). Thus, the effect of depletion was assessed on a murine melanoma model (B16). Three mouse groups were inoculated with B16 murine melanoma cells. Previously, a group was treated with αCD25, a second group was treated with isotype control rat IgG1 antibody (YCATE), and a third group was treated with isotonic saline solution. Tumor size was measured. As shown in the **Supplementary Figure 6**, tumor size was significantly smaller in αCD25-treated mice than in controls.

### Immune Response After Treg Depletion

**Figure 4:**
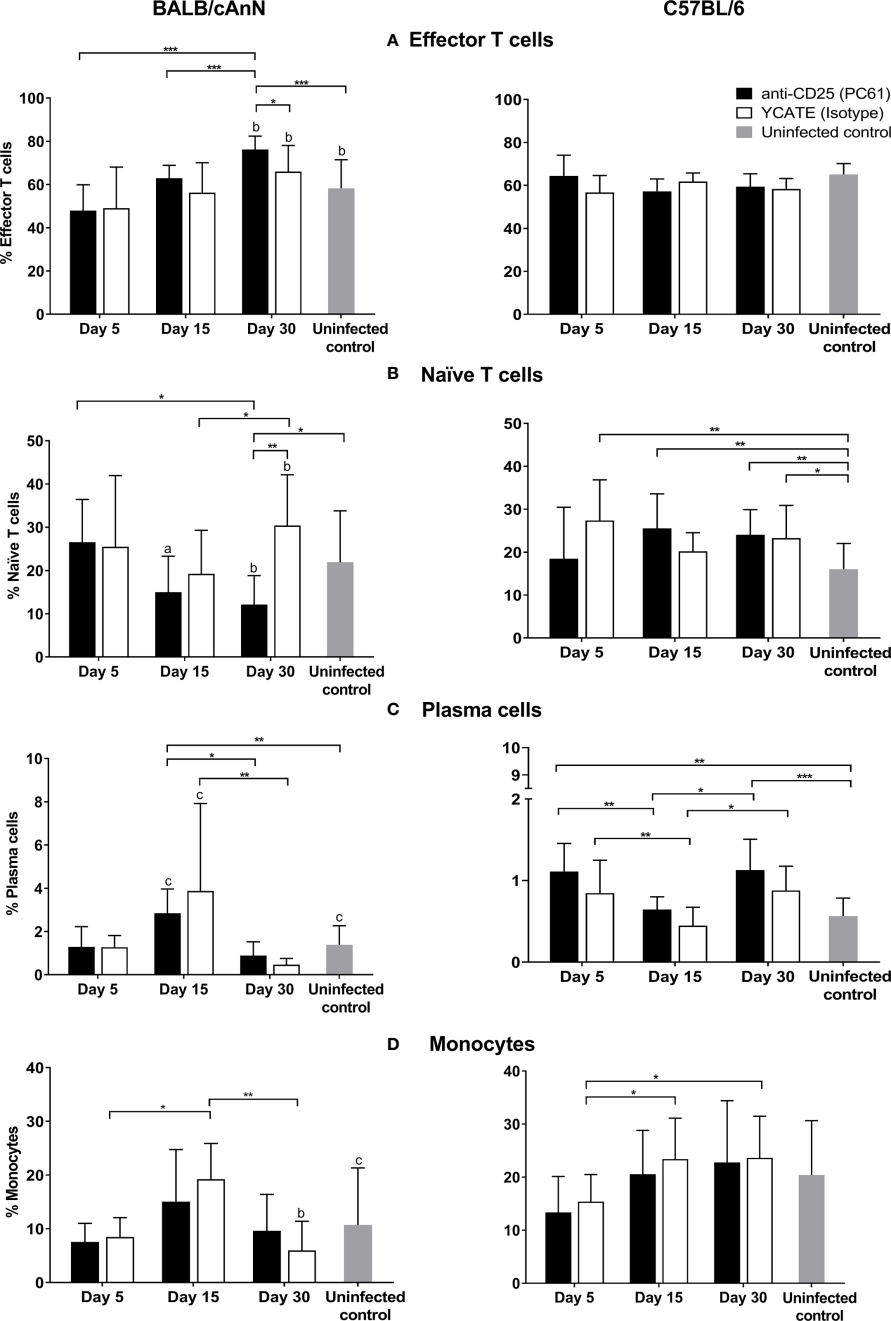
Effect of regulatory T cell depletion on the kinetics of various cell populations in peritoneum after *Taenia crassiceps* infection in Treg-depleted and non-depleted mice. **(A)** Effector T cells, **(B)** naïve T cells, **(C)** plasma cells, and **(D)** monocytes. Flow cytometry results are reported as a mean and standard deviation (bar graph). Six infected mice for each time (days 5, 15, and 30) and each condition (isotype or depleted) were included, as well as six non-infected animals of each strain. Significant differences in cell percentages as determined by the U Mann-Whitney test are shown as *P < 0.05, **P < 0.01, ***P < 0.005. Significant differences between strains were determined by the U Mann-Whitney test. Superscript letters indicate: ^a^ *P* < 0.05, ^b^ **P < 0.01, and ^c^ **P < 0.005.

Once Treg cells were proved to be functional, we decided to analyze the role of Treg depletion in BALB/cAnN and C57BL/6 mice during parasite infection. Various immune cell phenotypes were determined (**Figure 4**) in the peritoneum. An increase in the levels of effector T cells (CD3+CD4+CD44^hi^CD62L^low^) was observed in αCD25-treated, susceptible mice over time, being significant after day 30. At this time, isotype-treated control mice showed significantly lower levels of effector cells. In contrast, no changes in effector T cell percentages were observed in Treg-depleted and non-depleted C57BL/6 mice. Naïve T cell (CD3+CD4+CD44^low^CD62L^hi^) percentages decreased significantly in susceptible, αCD25-treated mice on day 30, while the percentage of these cells was higher in isotype-treated control mice. The percentage of plasma B cells and monocytes showed a peak on day 15 in susceptible mice, both in αCD25-treated and isotype-treated controls. In non-susceptible mice, plasma B cell percentages increased with respect to non-infected controls both in αCD25-treated mice and isotype-treated controls. No other analyzed population showed significant changes after Treg depletion (**Supplementary Figure 7**).

## Discussion

Parasites have co-evolved with their hosts and have acquired the ability to promote conditions that favor their establishment and survival. There are several examples of parasites profiting from host-derived molecules and resorting to evasion strategies to survive even in an immunologically specialized microenvironment like the central nervous system (CNS) (Adalid-Peralta et al., 2018).

In this work, we propose that *T. crassiceps* may promote the differentiation of regulatory T cells as a mechanism for immune evasion. Initially, we observed the percentage of immune populations in susceptible (BALB/cAnN) and non-susceptible (C57BL/6) mice. We analyzed the lymph nodes proximal to the parasite establishment site (peritoneum), since the immune response starts in the lymph nodes. In susceptible mice, Treg percentages were significantly increased on days 30, 90, and 130 post-infection, while Treg percentages in non-susceptible mice were increased only on day 5, and the percentage of activated CD127+ cells was increased at the same time. These results suggest that an immune response, including activated CD127+ and regulatory cells, could have been induced in non-susceptible mice on day 5. In contrast, in susceptible mice we only observed increased Treg percentages at early and late infection times, suggesting that a predominantly regulatory response is induced in this strain.

With respect to the infection site, the peritoneum, we observed that the percentage of activated CD127+ cells decreased on day 30 post-infection in non-susceptible mice. C57BL/6 mice have been reported to mount an effective immune response and be able to eliminate the parasite (Ramirez-Aquino et al., 2011); in fact, some mice of this strain failed to become infected in our study. The percentage of activated CD127+ cells on day 30 could be associated to parasite elimination and the restoring of immune homeostasis. In this strain, we observed a direct correlation between parasite load and activated CD127+ cell percentage, suggesting that parasites drive the activation of immune response. In contrast, in susceptible (BALB/cAnN) mice, the percentage of activated CD127+ cells are lower in early and late infection times, while Treg percentage are increased on days 5, 30, and 130. Thus, the parasite could be recruiting Tregs into the infection site to favor its establishment and survival.

In regard to parasite load, it should be noted that all mice of the susceptible strain were infected, in contrast to non-susceptible mice. The difference in parasite load values between both strains could be explained (at least partially) by a suppression of the effector immune response by Tregs in susceptible mice, since an inverse correlation between the levels of activated CD127+ cells and those of Tregs was observed only in the peritoneum of susceptible mice.

On the other hand, the presence of Tregs in the susceptible strain at early infection stages (5 days), when parasite load is low, could be explained by the fact that parasite antigens are abundantly present at the onset of the infection. These antigens could induce macrophages alternately activated, and these, in turn, induce Tregs cells. Indeed, it has been demonstrated that cysticerci drive antigen-presenting cells to promote Treg differentiation (Terrazas et al., 1998; Adalid-Peralta et al., 2013).

With respect to the immune proliferative response in infected susceptible and non-susceptible mice, a very modest response was observed against both specific and non-specific antigens. This result is not unexpected, as cysticerci have been reported to depress the proliferative response in both strains (Sciutto et al., 1995; Peón et al., 2013). In this study, we determined the proliferative response mediated by total cells, CD4+ T cells, effector T cells, and Tregs. The rationale for this analysis was the hypothesis that cysticerci could promote Treg cell proliferation, and in turn Tregs could suppress effector cell proliferation. This hypothesis relies on the finding that cysticerci promote Treg differentiation both *in vitro* and *in vivo* (Adalid-Peralta et al., 2012; Adalid-Peralta et al., 2013), and on the suppression mechanisms reported for Tregs (Vignali et al., 2008; Arce-Sillas et al., 2016). We only observed Treg proliferation in susceptible mice when spleen cells were stimulated with *T. crassiceps* antigen. The suppression of the immune response in susceptible mice could be associated (at least in part) to Tregs and other factors already described as parasite-secreted molecules and a decrease in the levels of IL-2 (Sciutto et al., 1995; Peón et al., 2013).

To deepen in the finding that a regulatory immune response predominated in susceptible mice at early infection times (5 and 30 days), we analyzed the relative expression of regulatory and pro-inflammatory molecules (**Figure 2**) in both strains. In non-susceptible mice, we found an overexpression of genes related to a pro-inflammatory immune response (IL-17F, IL-2, and PGR), while IL-4 was the only anti-inflammatory cytokine found overexpressed. The lower expression levels of pro and anti-inflammatory molecules in non-susceptible mice could be due to their specific microbiome, developed under conventional husbandry conditions. This is particularly relevant considering the reported differences in the microbiota from animals with a different genetic background (Campbell et al., 2012).

On the other hand, in susceptible mice, genes related to both proinflammatory and regulatory molecules were overexpressed (**Figure 2**). The main difference between strains is the expression of regulatory molecules on day 30 in susceptible mice. We identified several molecules that could be involved in the suppressive effect of Tregs. The higher expression of IL-10 and TGFβ suggests a cytokine-mediated suppression. The presence of CTLA4, IDO, and PDL1 and -2 also suggests suppression by cell-to-cell contact. Furthermore, the presence of CD39 and CD73 could be related to metabolic disruption *via* adenosine. The expression of granzyme A and perforin suggests suppression by cytolysis of effector cells. Other molecules like FAS, GILZ, TRAIL, and PDL1 and -2 have been related to suppression by Tregs (Hamdi et al., 2007; Kitazawa et al., 2007; Ren et al., 2007; Strauss et al., 2009; Calmette et al., 2014). Nevertheless, the results of genetic analyses in this study should be taken with caution, since we only collected peritoneal cells and there could be other cell types that favor the control of the immune response by the suppressive mechanism proposed, including alternatively activated macrophages, as previously described in *T. crassiceps* infection (Terrazas et al., 2005).

Finally, to better understand the role of Tregs in *T. crassiceps* infection, we performed depletion assays in the susceptible and non-susceptible mouse strains. Treg depletion before infection in susceptible mice led to a significant decrease in parasite load, along with higher parasite damage (degradation, calcification, and death). Treg removal could allow the host to mount an effective immune response and eliminate the parasite. In fact, 30 days after infection we observed significantly increased the percentage of effector cells in Treg-depleted mice, suggesting that Tregs participate in the suppression of the immune response against the parasite. In contrast, in non-susceptible mice, Treg depletion failed to modify the parasite load, and the immune response mediated by plasma cells was altered (**Figure 4**), suggesting that Tregs are not involved in controlling the effector response, which promotes parasite elimination. Thus, it is likely that the role of Tregs in this strain is homeostatic. However, to probe indirectly the functionality of Tregs in non-susceptible mice, we tested the effect of Treg depletion on tumor establishment and growth. In Treg-depleted mice, we observed significantly lower tumor growth rates; this is consistent with several studies reporting that Treg removal delayed tumor growth and improved the immune response (Onizuka et al., 1999; Nagai et al., 2004; Matsushita et al., 2008) and confirms that Tregs have a suppressive function in this strain. Overall, our data suggests a differential role of Tregs in two mice strains, begin beneficial for the parasite in the susceptible strain.

In conclusion, in the susceptible mouse strain, Treg cells play a key role in the ability of the parasite to infect an immunocompetent host. Tregs suppress the immune response through several of the mechanisms herein observed. In fact, the presence of Tregs in the site of infection stresses their role to favor parasite establishment and survival.

Tregs are not involved in susceptibility to infection in C57BL/6 mice but play a role in controlling the immune response against the parasite in the BALB/cAnN strain.

## Data Availability Statement

The datasets presented in this study can be found in online repositories. The names of the repository/repositories and accession number(s) can be found below: Flowrepository, FR-FCM-Z36M and https://figshare.com/articles/dataset/RT-PCR_DATA_Pro_and_anti-inflammatory_assay_panel_xls/14178281.

## Ethics Statement

The animal study was reviewed and approved by Scientific Committee and the Internal Committee for the Use and Care of Laboratory Animals (Permit No. 14/16) of the Instituto Nacional de Neurología y Neurocirugía.

## Author Contributions

LA-P: Conceived the study, designed the experiments, analyzed results, and drafted the manuscript. AL-R: Performed assays to quantify immune populations during infection and follow-up, performed proliferation assays; performed statistical analysis for all results. CC-V: Assessed *T. crassiceps* infection, maintained the parasite and performed all flow cytometry determinations. MN-O: Designed and performed the experiments to analyze immune populations by flow cytometry. MM-S: Designed and performed proliferation assay experiments. AG-S: Performed Treg depletion experiments *in vivo*, experiments with tumor cells and performed statistical analysis. VM-R: Participated in Treg depletion assays and tumor cell assays. DL-R: Contributed to Treg depletion and tumor cell assays; she maintained cysticercus stock in mice. EO-H: Participated in experiments of Treg depletion. JD: Provided anti-CD25 and isotype control antibodies to perform Treg depletion assays; designed Treg depletion experiments. JV-P: Designed, performed, and analyzed PCR assays. NR-M: Designed and performed proliferation assay experiments, participated in database construction. AA-S: Performed molecular biology experiments, analyzed results, and performed statistical analysis. SG-F: Participated in obtaining immune cells for all experiments. RP: Designed Treg depletion experiments, analyzed results, and drafted and approved the final manuscript. GF: Helped to conceive the study, designed experiments, analyzed results, and approved the final manuscript. ES: Designed experiments to demonstrate the presence of Tregs during parasite infection, analyzed results, and approved the final manuscript. ES-R: Designed the study protocol and performed assays to determine gene expression profile with the Fluidigm platform. Drafted and approved the final manuscript. All authors contributed to the article and approved the submitted version.

## Funding

Institutional foundings: INER (This work was partially supported by instances of the Mexican Government: Programa Presupuestal P016, Anexo 13 del Decreto del Presupuesto de Egresos de la Federación), and INNN, CONACYT (CB-2008-01 100708).

## Conflict of Interest

The authors declare that the research was conducted in the absence of any commercial or financial relationships that could be construed as a potential conflict of interest.
