# Supplementary material for: Regulatory T Cells as an Escape Mechanism to the Immune Response in Taenia crassiceps Infection
Source: Front Cell Infect Microbiol. 2021 Apr 13;11:630583. doi: 10.3389/fcimb.2021.630583 (PMC8076859; doi:10.3389/fcimb.2021.630583)
Supplement: Supplementary file 1 [file DataSheet_1.docx]

Supplementary Material

**Supplementary Table 1.** Pro-inflammatory, anti-inflammatory, and housekeeping genes.

| **Gene** | **Description** | **NCBI Reference Sequence** | **Efficiency**  **(%)*** |
| --- | --- | --- | --- |
| **Pro-inflammatory** | | |  |
| COX2 | *Mus musculus* mRNA for prostaglandin synthase cyclooxygenase, 3'UTR | NM_011198.3 | 90.54 |
| IFN-γ | *Mus musculus* interferon gamma (Ifng), mRNA | NM_008337.3 | 96.12 |
| IL-12b | *Mus musculus* interleukin12b (IL12b), protein code | NC_000077.6 | 100.00 |
| Il-17F | *Mus musculus* interleukin 17F (Il17f), mRNA | NM_145856.2 | 100.00 |
| IL-1b | *Mus musculus* interleukin 1 beta (Il1b), mRNA | NM_008361.3 | 100.00 |
| IL-2 | *Mus musculus* interleukin 2 (Il2), mRNA | NM_008366.3 | 100.00 |
| IL-6 | *Mus musculus* interleukin 6 (Il6), mRNA | NM_031168.1 | 111.35 |
| PGER2 | *Mus musculus* prostaglandin E receptor 2 (subtype EP2) (Ptger2), mRNA | NM_008964.4 | 112.33 |
| PGER4 | *Mus musculus* prostaglandin E receptor 4 (subtype EP4) (Ptger4), transcript variant 2, mRNA | NM_008965.1 | 107.19 |
| PGES2 | *Mus musculus* prostaglandin E synthase 2 (Ptges2), mRNA | NM_133783.2 | 94.14 |
| TNF | *Mus musculus* tumor necrosis factor (Tnf), mRNA | NM_013693.2 | 101.35 |
| **Anti-inflammatory** | | |  |
| CD39 | *Mus musculus* ecto-apyrase CD39 mRNA, complete cds | AF037366.1 | 102.79 |
| CD73 | *Mus musculus* 5' nucleotidase, ecto (Nt5e), mRNA | NM_011851.4 | 109.17 |
| CTLA-4 | *Mus musculus* cytotoxic T-lymphocyte-associated protein 4 (Ctla4), mRNA | NM_009843.3 | 111.48 |
| DAXX/FAS | Daxx Fas death domain-associated protein, protein code | NC_000083.6 | 112.31 |
| FASL | *Mus musculus* Fas ligand (TNF superfamily, member 6) (Fasl), transcript variant 1, mRNA | NM_010177.4 | 100.00 |
| EBI3 | *Mus musculus* Epstein-Barr virus induced gene 3 (Ebi3), mRNA | NM_015766.2 | 87.41 |
| IL-12a | *Mus musculus* interleukin 12a (Il12a), transcript variant 2, mRNA | NM_008351.2 | 119.25 |
| GITR V1 | *Mus musculus* tumor necrosis factor receptor superfamily, member 18 (Tnfrsf18), transcript variant 1, mRNA | NM_009400.2 | 83.06 |
| GRANZYME a | *Mus musculus* granzyme A (Gzma), mRNA | NM_010370.2 | 114.56 |
| GRANZYME b | *Mus musculus* granzyme B (Gzmb), mRNA | NM_013542.2 | 108.88 |
| IDO1 | *Mus musculus* indoleamine 2,3-dioxygenase 1 (Ido1), mRNA | NM_008324.1 | 100.00 |
| IL-4 | *Mus musculus* interleukin 4 (Il4), transcript variant 1, mRNA | NM_021283.2 | 100.00 |
| IL-10 | *Mus musculus* interleukin 10 (Il10), mRNA | NM_010548.2 | 100.00 |
| LAG-3 | *Mus musculus* lymphocyte-activation gene 3 (Lag3), mRNA | NM_008479.2 | 85.88 |
| PD1 | *Mus musculus* programmed cell death 1 (Pdcd1), mRNA | NM_008798.2 | 87.90 |
| PDL1 | *Mus musculus* CD274 antigen (Cd274), mRNA | NM_021893.3 | 102.03 |
| PDL2 | *Mus musculus* programmed cell death 1 ligand 2 (Pdcd1lg2), mRNA | NM_021396.2 | 94.96 |
| PRF1 | *Mus musculus* perforin 1 (pore forming protein), protein coding | NC_000076.6 | 100.00 |
| TGF-β1 | *Mus musculus* transforming growth factor, beta 1 (Tgfb1), mRNA | NM_011577.1 | 87.78 |
| TGF-β2 | *Mus musculus* transforming growth factor, beta 2 (Tgfb2), mRNA | NM_009367.3 | 102.50 |
| TRAIL | TNF-related apoptosis-inducing ligand, protein code | NC_000069.6 | 97.08 |
| VIP | *Mus musculus* vasoactive intestinal polypeptide (Vip), mRNA | NM_011702.2 | 100.00 |
| Housekeeping gene | | |  |
| GAPDH | *Mus musculus* glyceraldehyde-3-phosphate dehydrogenase, protein coding | NC_000072.6 | 102.31 |


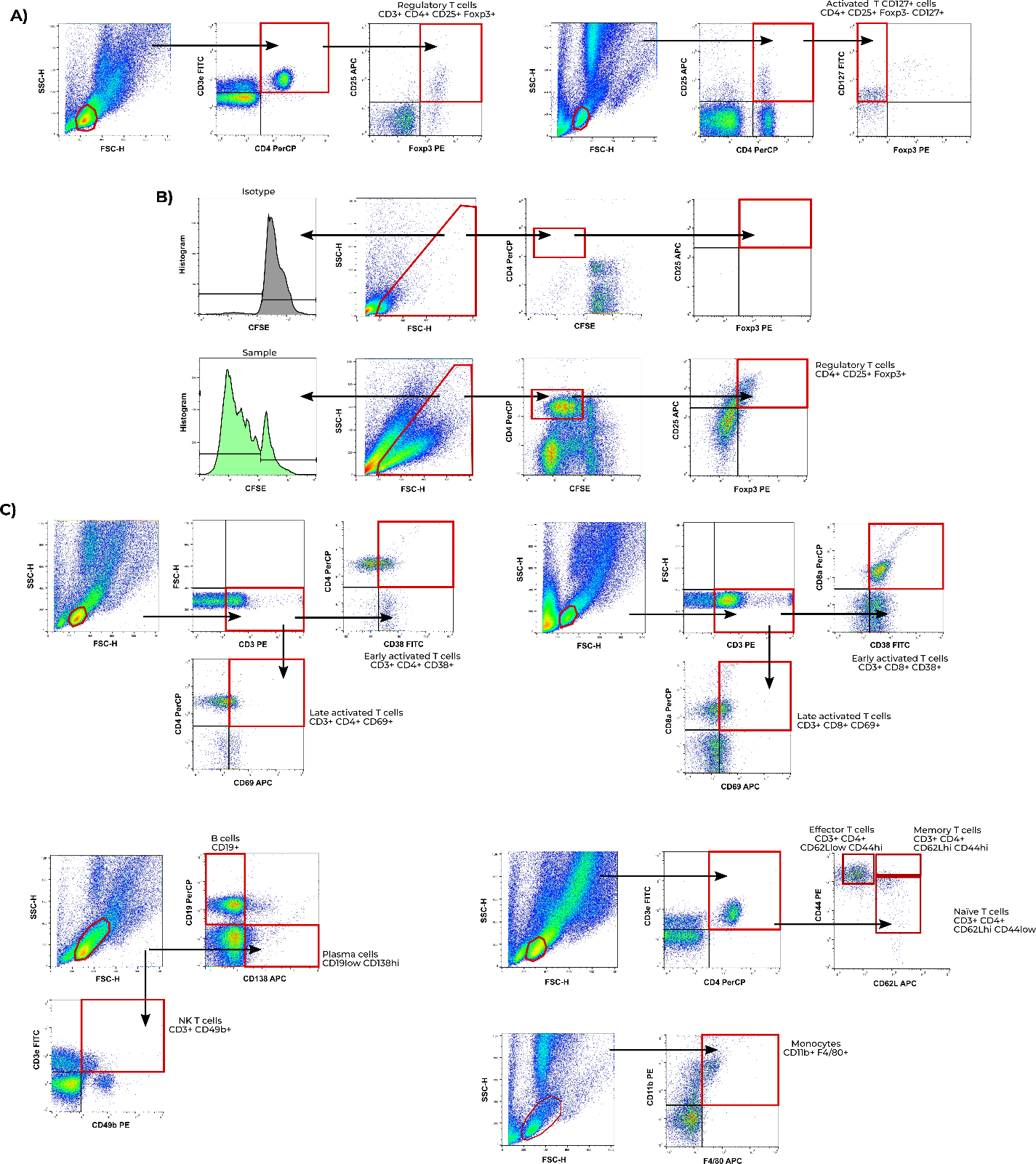


**Supplementary Figure 1. Phenotypic characterization of cell populations.** A) Representative example of Treg (CD4+CD25+FOXP3+) and activated CD127+ T cells (CD4+CD25+FOXP3−CD127+). B) Representative example of proliferation of Tregs (CD4+CD25+FOXP3+). C) Representative example of naïve T cells (CD3+CD4+ CD62L^hi^CD44^low^), effector T cells (CD3+CD4+CD62L^low^CD44^hi^), memory T cells (CD3+CD4+CD62L^hi^CD44^hi^), early activated CD4 (CD3+CD4+CD69+) or CD8 (CD3+CD8+CD69+) T cells, late activated CD4 (CD3+CD4+CD38+) or CD8 (CD3+CD8+CD38+) T cells, B cells (CD19+), plasma cells (CD19^low^CD138^hi^), NK T cells (CD49b+CD3+), and monocytes (CD11b+F4/80+).


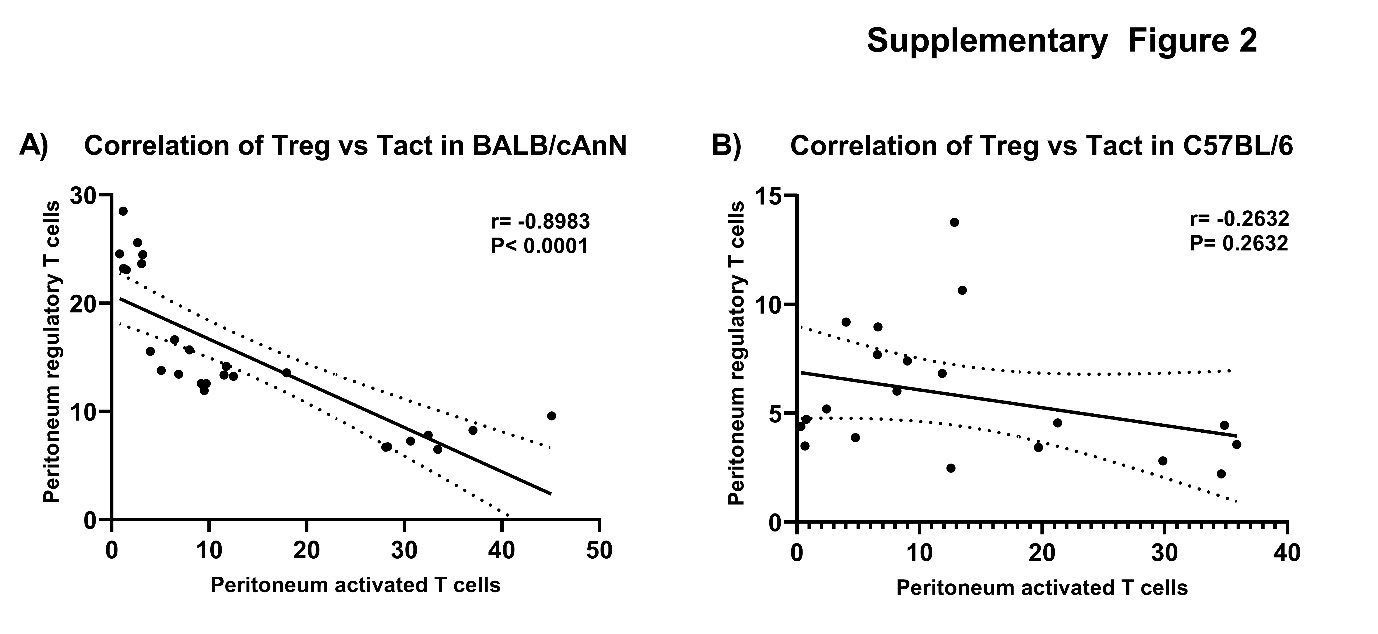


**Supplementary Figure 2.** Correlation between the levels of Treg cells and those of activated CD127+ cells from peritoneum. A) BALB/cAnN and B) C57BL/6 mice. In total, 24 infected mice of each strain (six for each time point) were analyzed. The Spearman’s correlation coefficient and *P*-value are shown on the plot.


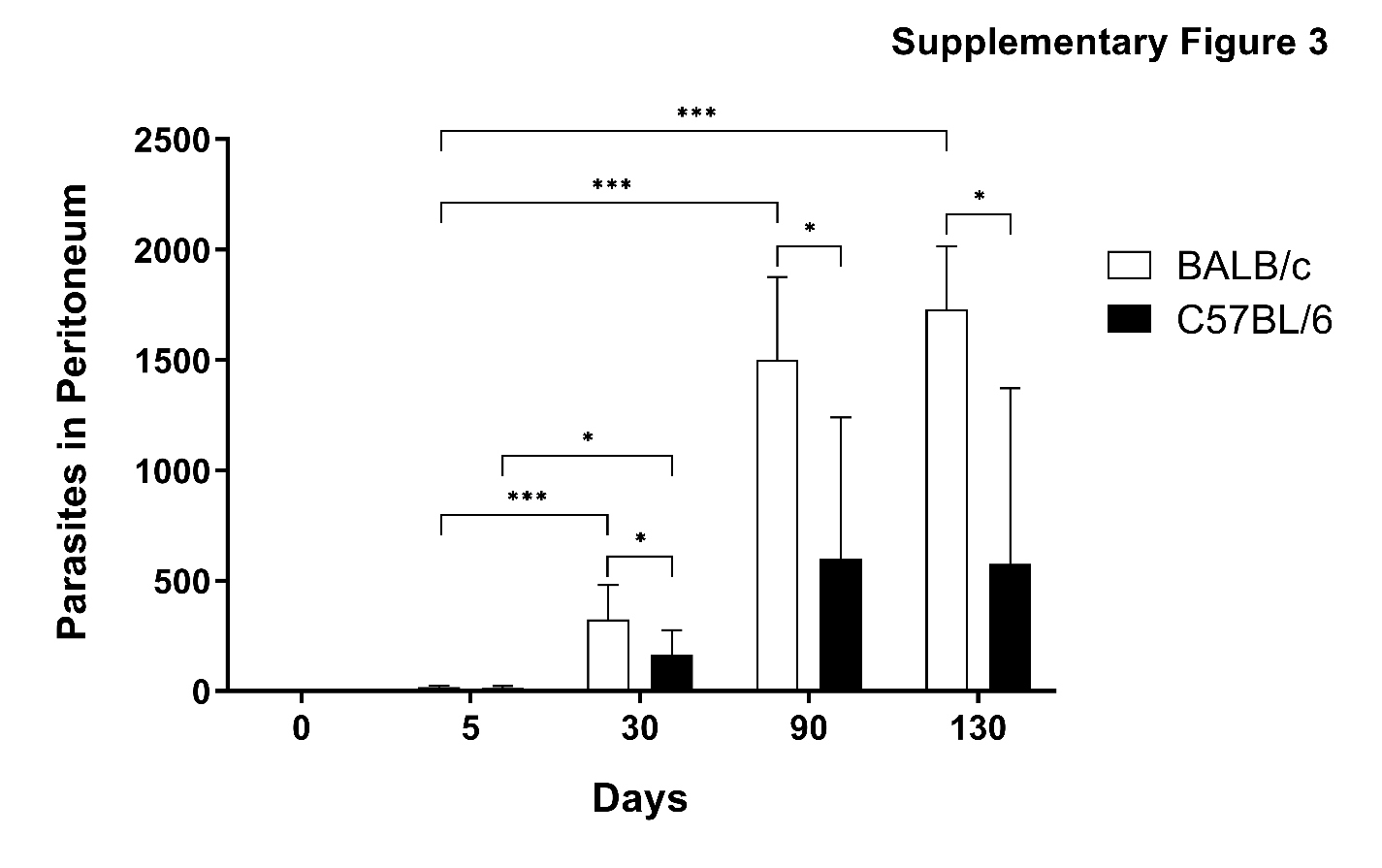


**Supplementary Figure 3.** Parasite number in the site of infection (peritoneum) in susceptible, BALB/cAnN and non-susceptible, C57BL/6 mice on day 5, 30, 90, and 130 post-infection. Six infected mice and 6 controls were included for each time point analyzed. Significant differences were determined by the U Mann-Whitney test. **P* < 0.05, ***P* < 0.01, ****P* < 0.005.

**Supplementary Table 2.** Percentage of immune CD4+ T cells and naïve-like cells in peritoneum, lymph nodes, and spleen of susceptible (BALB/cAnN) and non-susceptible (C57BL/6) mice.

| **Day post-infection** | | | | | |
| --- | --- | --- | --- | --- | --- |
| **Cell phenotypes** | **Uninfected controls** | **5** | **30** | **90** | **130** |
| **T CD4 cells** | **PERITONEUM** | | | | |
| BALB/cAnN | 8.47±5.80 | 11.76±4.97 | 10.79±4.57 | 13.85±3.54** | 6.25±3.43 |
| C57BL/6J | 13.24±6.90 | 21.71±4.80***^b^ | 21.29±4.96**^c^ | 12.37±5.76 | 18.98±3.21**^c^ |
| **Naïve-like cells^Ω^** |  |  |  |  |  |
| BALB/cAnN | 52.74±19.34 | 16.57±2.95*** | 29.14±9.16 | 36.15±13.69 | 6.38±2.42*** |
| C57BL/6J | 86.59±7.34 | 86.46±6.09^c^ | 95.36±1.47***^c^ | 92.79±2.51*^b^ | 93.57±1.84^a^ |
| **CD4 T cells** | **LYMPH NODES** | | | | |
| BALB/cAnN | 44.22±9.98 | 45.35±10.03 | 41.81±15.26 | 43.10±5.11 | 41.75±4.70 |
| C57BL/6J | 34.50±4.89 | 36.00±3.15 | 35.17±3.52 | 32.98±2.67^c^ | 30.30±4.36*^b^ |
| **Naïve-like cells^Ω^** |  |  |  |  |  |
| BALB/cAnN | 39.41±25.97 | 15.82±9.80 | 37.32±25.78 | 35.23±11.22 | 20.56±6.18 |
| C57BL/6J | 89.72±6.90 | 90.18±1.94^a^ | 92.94±2.13^b^ | 87.08±6.21^b^ | 89.30±4.25*^a^ |
| **CD4 T cells** | **SPLEEN** | | | | |
| BALB/cAnN | 32.60±4.77 | 28.25±2.99* | 25.60±1.50*** | 36.41±1.64* | 30.27±3.54 |
| C57BL/6J | 24.60±5.24 | 16.69±2.67***^c^ | 25.32±4.82 | 29.62±4.03**^b^ | 18.16±4.83**^c^ |
| **Naïve-like cells^Ω^** |  |  |  |  |  |
| BALB/cAnN | 35.52±11.87 | 22.66±9.81* | 36.89±8.84 | 18.47±3.30*** | 21.54±4.18*** |
| C57BL/6J | 84.66±6.71 | 79.90±3.99^b^ | 92.88±1.30***^b^ | 93.54±2.83**^b^ | 79.46±9.78^a^ |

^Ω^Naïve-like lymphocytes (CD4+CD25-Foxp3-CD127+). Comparison at each time with non-infected controls. Six infected mice and 6 controls were included for each time point analyzed. **P* < 0.05, ***P* < 0.001, ****P* < 0.0001. Comparison between mouse strains. ^a^ *P* < 0.05, ^b^ *P* < 0.001, and ^c^ *P* < 0.0001.


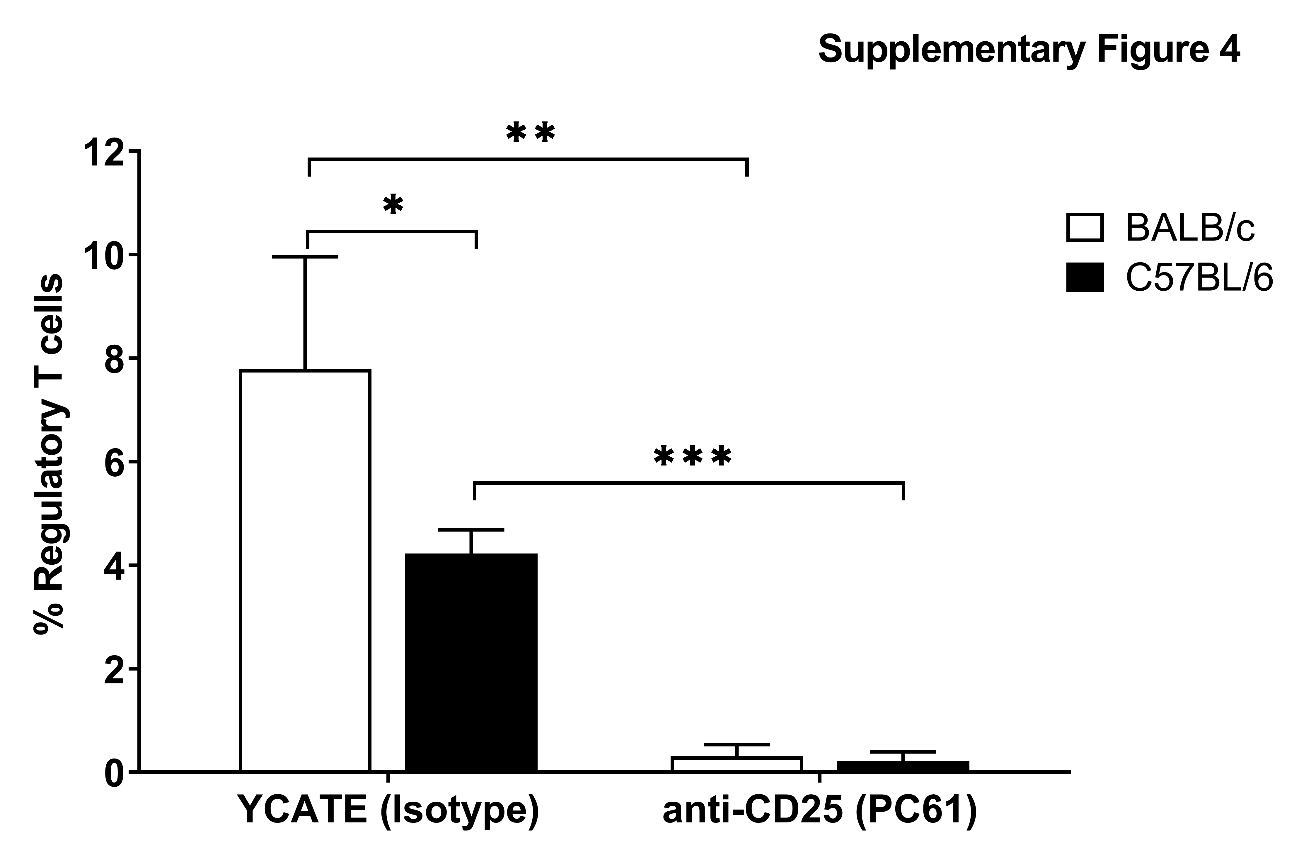


**Supplementary Figure 4.** Percentage of regulatory T cells in the peritoneum at the time of infection (day 0) in susceptible BALB/cAnN and non-susceptible C57BL/6 mice after either anti-CD25 (PC61) or isotype (YCATE) treatment. Four mice were used for each condition analyzed. Flow cytometry results are reported as a mean and standard deviation (bar graph). Significant differences in cell percentages as determined by the U Mann-Whitney test are shown as * *P* < 0.05, ***P* < 0.01, ****P* < 0.005.


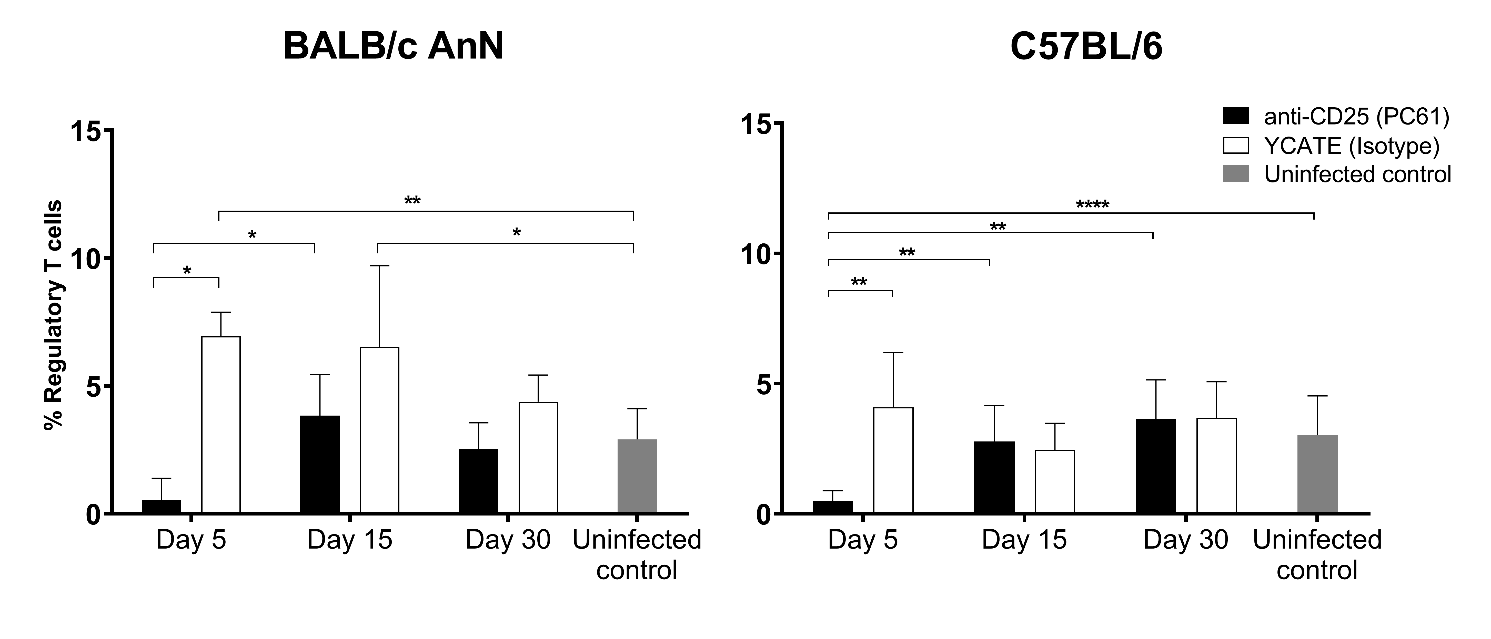


**Supplementary Figure 5.** Percentage of regulatory T cells in the peritoneum on day 5, 15, and 30 after infection in susceptible BALB/cAnN and non-susceptible C57BL/6 mice treated with either anti-CD25 (PC61) or isotype (YCATE). Six infected mice for each time (days 5, 15, and 30) and each condition (isotype or depleted) were included, as well as six non-infected animals of each strain. Flow cytometry results are reported as a mean and standard deviation (bar graph). Significant differences in cell percentages as determined by the U Mann-Whitney test are shown as **P* < 0.05, ***P* < 0.01, ****P* < 0.005.


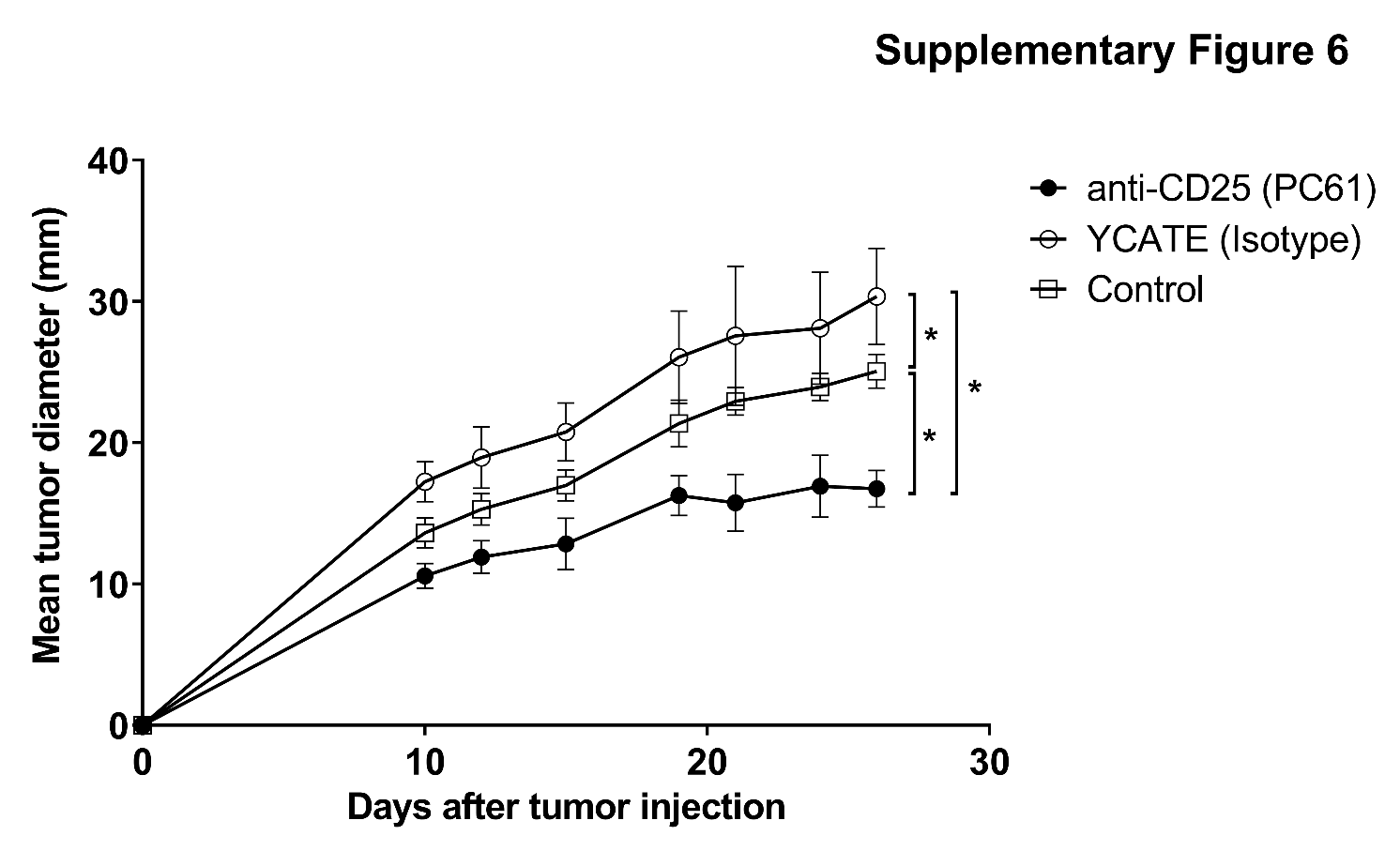


**Supplementary Figure 6.** Treg removal allows to mount an anti-tumor response in the non-susceptible strain. Melanoma B16 cells (2 $\times$ 10^5^) were subcutaneously injected in C57BL/6 mice treated with either anti-CD25 (PC61) or isotype (YCATE) two days before tumor inoculation. Tumor size was measured as the product of the greatest longitudinal diameter (length) $\times$ greatest transverse diameter (width). Six infected mice for each group were analyzed. Significant differences were determined by the Wilcoxon signed-rank test. **P* < 0.05.


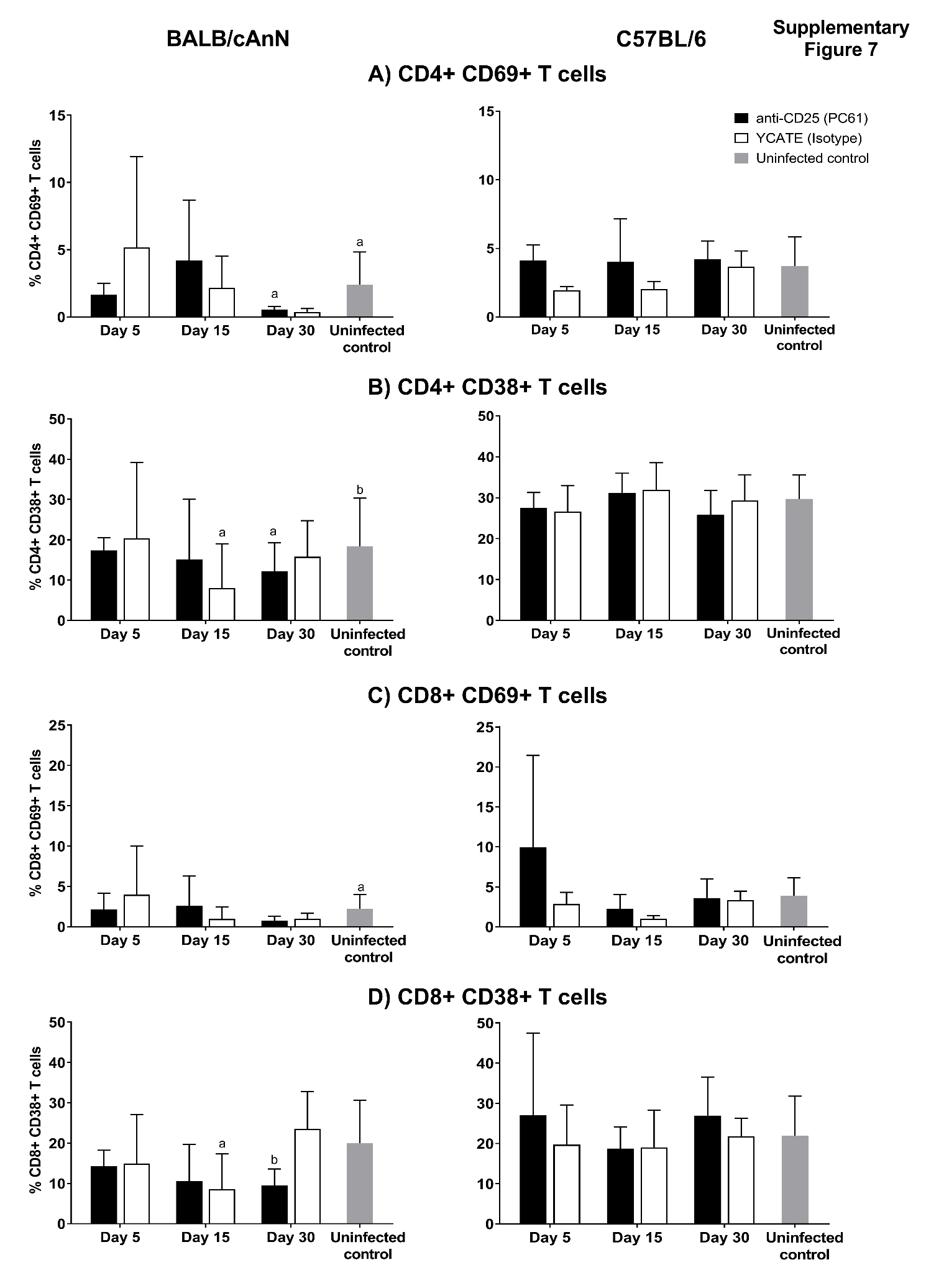


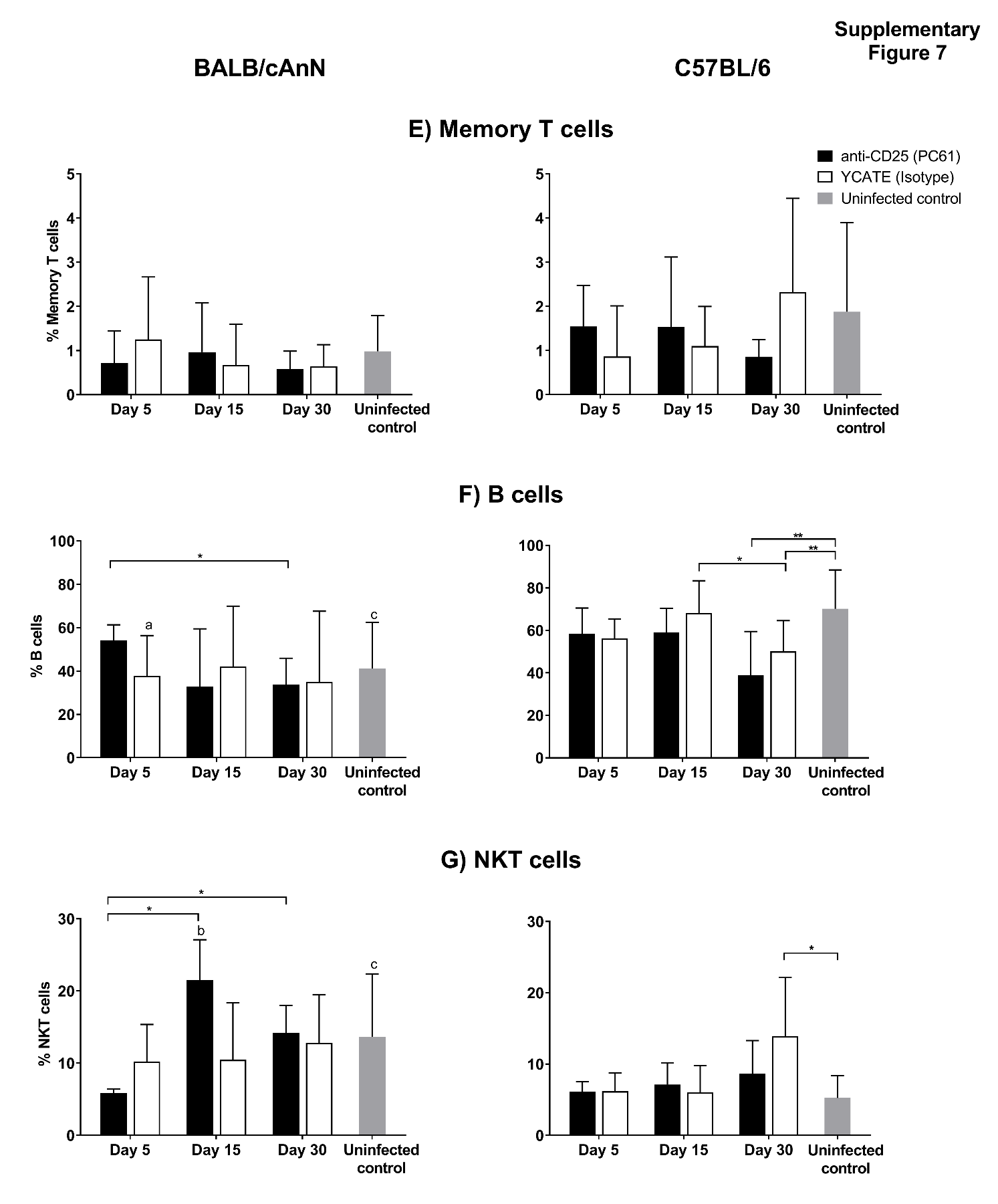


**Supplementary Figure 7.** Kinetic of immune populations in susceptible BALB/cAnN and non-susceptible C57BL/6 mice treated with either anti-CD25 (PC61) or isotype (YCATE). A) Early activated CD4+ cells, B) late activated CD4+ cells, C) early activated CD8+, D) late activated CD8+, E) memory T cells, F) B cells, G) NKT cells. Six infected mice for each time (days 5, 15, and 30) and each condition (isotype or depleted) were included, as well as six non-infected animals of each strain. Flow cytometry results are reported as a mean and standard deviation (bar graph). Significant differences in cell percentages as determined by the U Mann-Whitney test are shown as **P* < 0.05, ***P* < 0.01. Significant differences between strains were determined by the U Mann-Whitney test. Superscript letters indicate: a P < 0.05, b **P < 0.01, and c **P < 0.005.
